# Identifying Interventions and Their Efficacy as Used by a Community Agency Managing and Responding to Elder Abuse

**DOI:** 10.1177/0733464821992606

**Published:** 2021-02-13

**Authors:** Jennifer E. Storey, Samantha Hart, Melanie R. Perka

**Affiliations:** 1 University of Kent, Canterbury, UK; 2 Royal Holloway University of London, Egham, UK; 3 Catholic Social Services, Edmonton, Alberta, Canada

**Keywords:** elder mistreatment, risk management, abuse of older adults, management strategy

## Abstract

Limited research has been conducted to identify how elder abuse (EA) can be managed and prevented. Interventions employed by a community agency multidisciplinary team across 164 EA cases were examined. Results identified the largest number (*N* = 369) and widest variety of EA interventions to date. Using content analysis, interventions with similar proximal goals were grouped into 30 intervention strategies to evaluate efficacy and 12 higher-order intervention categories to guide practice. Intervention outcomes were rated as positive, negative, neutral, could not implement, or unknown. Positive outcomes were the most common (35%), and also included novel and/or effective interventions aimed at perpetrators such as physical treatment, social support, and communication. Few (1%) interventions had negative outcomes. Many interventions could not be implemented (21%), often due to a lack of funding or victim refusal. Results suggest changes to policy, practice, and research methodology, which could increase positive outcomes through facilitation of intervention implementation and improved data access.

Elder abuse (EA), or the abuse of older adults, is recognized as a serious public health concern affecting a growing older adult population (Centers for Disease Control and Prevention [CDCP], 2007; Yon et al., 2017). Research examining risk management to combat this problem is limited but necessary to identify the interventions used and their efficacy (Fearing et al., 2017).

EA can be defined as “a single or repeated act, or lack of appropriate action, occurring within any relationship where there is an expectation of trust that causes harm or distress to an older person” (Action on Elder Abuse, 1995, p. 11) and includes five types of abuse: physical, sexual, psychological/emotional, financial, and neglect (Wolf et al., 2002). Elder self-neglect is a separate form of self-inflicted harm that refers to the “refusal or failure to provide oneself with care” (Dong, 2017, p. 949). A meta-analysis of studies from 28 countries found a pooled past-year prevalence of EA of 15.7% (Yon et al., 2017) and a growing older adult population will require increased risk management (CDCP, 2007). The consequences of EA and insufficient risk management on victims are substantial and include physical injury/death, mental deterioration, and financial loss (Dong et al., 2009; World Health Organization, 2016).

To avoid these devastating consequences and address EA, effective risk management is required. Risk management is the process of planning and implementing strategies/interventions to prevent violence (Viljoen et al., 2018). Multiprofessional teams are widely recommended for EA management and in need of evaluation (Pillemer et al., 2016).

Risk management has received little attention in the EA literature (Baker et al., 2017); it has been, however, identified as the most pressing need within the field (Pillemer et al., 2016; Roberto, 2016). Recently, five promising studies have examined interventions involving wider programs, or multidisciplinary interventions for EA. As a result, there is a growing, but still incomplete, understanding of what interventions are utilized by EA services to manage cases and which interventions are effective (Baker et al., 2017; Fearing et al., 2017).

First, Alon and Berg-Warman (2014) examined the Specialized Unit for the Prevention and Treatment of Elder Abuse in Israel. Questionnaires and interviews were used to identify seven management interventions and assess the unit. Results showed significant improvements in cases of neglect, with legal interventions and social service interventions improving outcomes in most cases.

Vladescu and colleagues (2000) assessed a case management program in Ontario, Canada. Eight management interventions directed at victims were identified and an examination of 26 client records showed improvements in over half of cases. Although a useful model of the program was provided, results were limited because only some of the interventions were identified and the program dealt only with victims who were competent.

Mariam and colleagues (2015) evaluated an intervention program in California. The researchers connected victims with community resources and implemented motivational interviewing to encourage service engagement. Results showed a significant decrease in risk factors, with almost two-thirds of victims progressing toward change. However, the interventions leading to these positive outcomes were not identified.

Rizzo and colleagues (2015) examined a social work–lawyer intervention model in New York. Findings from the multisite random sample of 250 cases showed that a reduction in abuse was associated with client retention, program fidelity, and exposure to multidisciplinary services. Results support the use of multidisciplinary models but did not investigate the impact of individual interventions.

In the study most like the present research, Nahmiash and Reis (2001) examined intervention plans used in a program in Montreal, Canada. Interventions undertaken in a subset of available cases (*n* = 83) were grouped into 10 categories. Categories were rated for success and client acceptance. Rehabilitative, homecare, and empowerment interventions were the most successful for and accepted by victims. Referrals to increase socialization were the least successful. For perpetrators, counseling for anxiety, stress, and depression, as well as education and training were the most successful. Results are limited by the study’s focus on caregivers alone. Furthermore, the interventions used with caregivers primarily support the caregiver stress model as an explanation for abuse and do not address other explanations and risk factors for EA that are more empirically supported (Pillemer & Finkelhor, 1989; Storey, 2020). Although greater detail is provided in this study compared with others, examples of the interventions used are generally not provided. Furthermore, efficacy data are only provided for two of eight intervention categories.

## Current Study

Despite the recent increase, research remains sparse, is often nonsignificant (Ploeg et al., 2009), and frequently examines only a subset of EA cases. For example, Ayalon and colleagues (2016) found that 19 of 24 studies reviewed examined only caregivers. Further research is very much needed (Fearing et al., 2017; Pillemer et al., 2016; Roberto, 2016). We are far from a clear understanding of what works to stop EA and likely have not even identified all the interventions being utilized. The present study builds on previous research by identifying and evaluating the efficacy of all risk management interventions recommended/implemented by a multidisciplinary program, from reporting to case conclusion. In identifying the interventions used with all types of perpetrators, this study aims to provide guidance related to the use of risk management across all EA cases by answering the following questions: (a) What interventions are being utilized in cases of EA? and (b) Are those interventions effective in the management of EA?

## Method

### Overview

As part of a program evaluation, a secondary analysis of 164 cases of EA reported to the Elder Abuse Resource and Supports Team (EARS) in Edmonton, Canada, was conducted. No contact was made with those individuals involved in the case. EARS is part of Catholic Social Services and is specialized in attending to cases of EA identified as low-moderate risk. EARS caseworkers consist primarily of registered social workers; some, however, have higher education backgrounds in related fields. Where cases are identified as high risk, they are managed in conjunction with partners from Edmonton Police Services, the City of Edmonton, the Victorian Order of Nurses, and Covenant Health—this group is called the Senior’s Protection Partnership (SPP). Reports of EA can be made to the EARS team by victims, other service providers, and concerned members of the public.

A representative sample, including all cases of EA reported to EARS and handled by EARS/SPP over a 27-month period, was examined. All risk management interventions (Table 1) recommended or implemented by EARS/SPP were recorded, as were their outcomes. For clarity, individuals thought to be engaging in abusive behavior toward an older adult will be referred to as *perpetrators*, and those experiencing abuse as *victims*.

**Table 1.** Risk Management Terminology and Frequency Across Cases.

Terminology	Definition	Example	Frequency
Risk management			
Intervention	An action suggested or taken by those involved in cases where the goal is to promote desistance of elder abuse or decrease the vulnerability of victims.	• Mental health crisis team referral • Homecare set up	369
Intervention strategy	A group of interventions that have the same proximal goal or achieve the same goal in a similar way.	• Mental health care • Physical health care	30
Intervention category	A collection of intervention strategies that target the same issue or individual.	• Victim care	12
Outcomes			
Positive	The intervention had a positive impact on the case.	• The cessation of abuse • The victim agreed to intervention • Obtained confirmation or admission of abuse • Victim is happy, with no abuse concerns	384
Neutral	The intervention had no identifiable impact on the case.	• There was no change in the victim’s circumstances • No change in abuse	177
Negative	The intervention had a negative impact on the case.	• The abuse worsened • The victim withdrew from services • The victim became more vulnerable	13
Could Not Implement (CNI)	The recommended intervention was blocked from being implemented by the victim, perpetrator, or another individual involved in the case	• The victim refused to limit contact with the perpetrator • The family did not call police for help as agreed	236
Unknown	The impact of an implemented intervention could not be identified.	• The case was closed prior to the tactic outcome being recorded • The victim or family did not follow-up to feedback as agreed	293

### Cases

**Figure 1.**
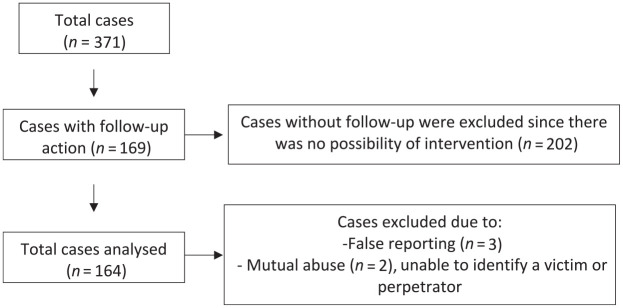
Exclusion process.

The cases reviewed constitute a representative normative sample of those handled by EARS/SPP, as all cases over a 27-month period were examined. A total of 164 cases were eligible for inclusion in the study (Figure 1). This sample was used in Storey and Perka (2018); there is, however, no overlap in the data presented. A total of 177 victims (range per case = 1–2) and 206 perpetrators (range per case = 1–4) were involved in the 164 cases reviewed. Victims were primarily female (70%, *n* = 124) with an average age of 75 (*SD* = 9.40, range = 50–95) years. Perpetrators were primarily victims’ adult-children (59%, *n* = 121) and had a mean age of 47 (*SD* = 15.84, range = 13–89) years (33% missing information).

### Materials

Management information was extracted from anonymized case files that included two types of documents, an intake form and case notes. Intake forms document the initial report of EA, including demographic information and information required for follow-up. Case notes record all interactions that caseworkers have with individuals involved in the case, including any interventions recommended/implemented. Case notes begin at the initial report of abuse and continue until case closure. Cases are closed when the competent victim or their appointed decision maker (who was not the perpetrator) no longer wants assistance (e.g., help is no longer needed, victim withdraws from intervention). Cases contained between one and 118 case notes (*M* = 15, *SD* = 17.35).

### Procedure

Anonymized case files were reviewed, each risk management intervention recommended or implemented therein was recorded along with its outcome. Five outcome types were identified and coded for each intervention (Table 1). To ensure reliability between the two raters (J.E.S. and S.H.), 20% of cases (*n* = 33) were coded by both raters and then indexed using interclass correlation coefficients (ICC_1_; two-way mixed effects model, absolute agreement method). ICC values compared the total number of management interventions identified in a case; reliability was excellent (ICC_1_ = .92, confidence interval [CI] = [.84, .96]; Cicchetti & Sparrow, 1981).

### Data Analysis

Given the high volume of management interventions identified, categorization was required for analysis and to facilitate comprehension. Like Nahmiash and Reis (2001), content analysis was used to create groupings as it is appropriate for exploratory research (Thomas, 2003). Content analysis, as per Erlingsson and Brysiewicz (2017), was completed by S.H. and reviewed and agreed upon by J.E.S. using the following steps: (a) cases were read and reread to identify meaning units; herein, meaning units were the actions taken to end EA; (b) meaning units were condensed to capture core content; herein, “interventions” units were identified and summarized to capture the core content of the action and a code was given that described the meaning—this was reviewed by J.E.S.; and (c) codes were grouped by shared meaning; herein, grouping was done at two levels, interventions with shared meanings were grouped to create “intervention strategies,” and, next, intervention strategies were grouped based on wider shared meanings, creating “intervention categories”—this was reviewed by J.E.S. (Table 1).

Results are reported for the two highest intervention levels (intervention strategy and intervention category) for several reasons. First, too many interventions (*n* = 369) were identified to report individually; second, this overwhelming level of detail would not have been useful for practitioners; and third, some interventions were case or jurisdiction specific. Thus, broader conceptual groups (strategies and categories) were developed to provide more generalizable guidance for practice. Intervention strategies are reported because they provide meaningful frequency data related to outcomes and are sufficiently detailed to guide practice. Intervention categories are reported as they identify a small number of key intervention targets that, due to their breadth, can be used as an aid memoire for practicing professionals.

Frequency analysis was conducted using SPSS Version 22. Ethical approval was granted by Royal Holloway University of London and permission for the study was obtained by the EARS/SPP program manager. Case files were collected and anonymized by a member of the EARS/SPP team who was paid for her time by Catholic Social Services. Researchers only reviewed anonymized information.

## Results

Across the 164 cases examined, 369 interventions were identified. Those interventions were recommended/implemented 1,103 times with a mean of seven interventions (*SD* = 4.84) and a range of zero to 26 interventions per case (one case had no interventions recommended/implemented). Thirty intervention strategies and 12 higher-order intervention categories were identified. Each of the 12 intervention categories is described below and listed in Table 2, with their encompassed intervention strategies and illustrative examples of the interventions used. Table 2 also provides the frequency of intervention use overall and the range of interventions used per case. A minority of intervention strategies (*n* = 4) were so unique that they could not be categorized, as such they are presented as stand-alone categories.

**Table 2.** The Frequency, Range, and Percent of Intervention Strategy and Category Use by Efficacy.

		*Outcome*					
*Category*	*Strategy*	*N* (range per case)	Positive	Negative	Neutral	CNI	Unknown
*Monitoring*		64 (0–4)	30 (47%)	0	11 (17%)	12 (19%)	11 (17%)
*Creating barriers to abuse*		86	58 (67%)	3 (3%)	6 (7%)	8 (9%)	11 (13%)
	Supervision	40 (0–3)	32 (80%)	1 (3%)	2 (5%)	4 (10%)	1 (3%)
	Removing Access To Methods Of Abuse	27 (0–2)	18 (67%)	1 (4%)	1 (4%)	1 (4%)	6 (22%)
	Reduced Contact	19 (0–4)	8 (42%)	1 (5%)	3 (16%)	3 (16%)	4 (21%)
*Perpetrator treatment*		56	18 (32%)	2 (4%)	7 (13%)	17 (30%)	12 (21%)
	Social Support	19 (0–3)	5 (26%)	0	2 (11%)	8 (42%)	4 (21%)
	Mental Health Treatment	25 (0–2)	8 (32%)	2 (8%)	2 (8%)	7 (28%)	6 (24%)
	Physical Treatment	9 (0–2)	5 (56%)	0	1 (11%)	1 (11%)	2 (22%)
	Substance Abuse Support	3 (0–2)	0	0	2 (67%)	1 (33%)	0
*Communicating with the perpetrator*		51 (0–3)	18 (35%)	2 (4%)	13 (25%)	8 (16%)	10 (20%)
*Improving access to support*		286	76 (26%)	2 (1%)	37 (13%)	74 (26%)	97 (34%)
	Legal Support	114 (0–6)	37 (32%)	1 (1%)	18 (16%)	24 (21%)	34 (30%)
	Resource Sharing	87 (0–4)	12 (14%)	0	12 (14%)	27 (31%)	36 (41%)
	Support With Tasks	30 (0–5)	8 (27%)	1 (3%)	3 (10%)	10 (33%)	8 (27%)
	Financial Support	24 (0–3)	11 (46%)	0	2 (8%)	3 (13%)	8 (33%)
	Support Groups	14 (0–2)	3 (21%)	0	1 (7%)	5 (36%)	5 (36%)
	Cultural And Religious Services	10 (0–1)	2 (20%)	0	1 (10%)	3 (30%)	4 (40%)
	Food Resources	7 (0–2)	3 (43%)	0	0	2 (28%)	2 (28%)
*General safety actions*		86 (0–4)	16 (19%)	0	15 (17%)	11 (13%)	44 (51%)
*Home safety*		65	20 (31%)	0	10 (15%)	28 (43%)	7 (11%)
	Housing Support	53 (0–2)	17 (32%)	0	7 (13%)	24 (45%)	5 (9%)
	Environmental Changes	12 (0–2)	3 (25%)	0	3 (25%)	4 (33%)	2 (17%)
*Education*		34	9 (27%)	0	12 (35%)	4 (12%)	9 (27%)
	Elder Abuse Education	30 (0–3)	7 (23%)	0	11 (37%)	4 (13%)	8 (27%)
	Mental Health And Substance Abuse Education	4 (0–2)	2 (50%)	0	1 (24%)	0	1 (25%)
*Victim care*		69	27 (39%)	1 (1%)	12 (17%)	16 (23%)	13 (19%)
	Physical Care	41 (0–2)	18 (44%)	0	6 (14%)	10 (24%)	7 (17%)
	Mental Health Care	27 (0–3)	9 (33%)	1 (4%)	5 (19%)	6 (22%)	6 (22%)
	Substance Abuse Care	1	0	0	1 (100%)	0	0
*Victim well-being*		110	35 (31%)	1 (1%)	23 (21%)	31 (28%)	20 (18%)
	Emotional Support	54 (0–5)	22 (41%)	0	7 (13%)	16 (30%)	9 (16%)
	Reasoning With Victim	21 (0–3)	4 (19%)	1 (5%)	9 (43%)	4 (19%)	3 (14%)
	Development Of Communication	35 (0–2)	9 (26%)	0	7 (20%)	11 (31%)	8 (23%)
*Capacity assessment*		18	10 (55%)	1 (5%)	3 (17%)	1 (5%)	3 (17%)
*Involvement of others*		178	67 (38%)	1 (1%)	28 (16%)	26 (15%)	56 (32%)
	Multidisciplinary Teams	110 (0–4)	41 (37%)	1 (1%)	19 (17%)	17 (15%)	32 (29%)
	Family And Friends	68 (0–4)	26 (38%)	0	9 (13%)	9 (13%)	24 (35%)
*Total*		1,103	384 (35%)	13 (1%)	177 (16%)	236 (21%)	293 (27%)

*Note*. CNI = could not implement.

### Monitoring

Monitoring interventions aimed to minimize risk through surveillance, including monitoring the victim and the perpetrator. Sixteen different types of monitoring interventions were identified, such as unannounced home visits and the victim attending a day care program so that other professionals had a chance to engage and observe the victim in a different setting.

### Creating Barriers to Abuse

The three intervention strategies in this category were designed to restrict the possibility of abuse occurring by increasing barriers to engaging in abusive behavior for the perpetrator. First, *Supervision* included 15 different interventions that placed legal restrictions on the perpetrator making it more difficult or consequential for them to reoffend. Example interventions included arrest, restraining order, and removal from home by police. Second, *Removing Access to Methods of Abuse* eliminated the means through which the perpetrator engaged in abuse. Nine such interventions were identified, all were financial, and included removing the perpetrator as the victim’s power of attorney and implementing a personal directive for the victim. Third, *Reduced Contact* included 12 victim-focused interventions that increased victim safety by ending or minimizing victim–perpetrator contact. Examples include blocking the perpetrator’s phone number and having the victim avoid places where they would commonly encounter the perpetrator.

### Perpetrator Treatment

This category included four intervention strategies involving the recommendation/implementation of services for the perpetrator to improve psychosocial deficiencies that may have contributed to abusive behavior. *Social Support* included 17 interventions designed to support the perpetrator in living a more independent pro-social life—this included providing support to obtain employment, benefits, or low-cost housing. *Mental Health Treatment* involved 12 interventions supporting the perpetrator’s Mental well-being, such as psychiatric medication and counseling. *Physical Treatment* included physical health care and treatment for the perpetrator. Four such interventions were employed, including inpatient treatment and placement in long-term care. *Substance Abuse Support* included five addiction-focused interventions such as the perpetrator attending addiction treatment programs and providing information regarding addiction support groups.

### Communicating With the Perpetrator

This strategy included eight perpetrator-focused interventions where issues related to the abusive dynamic were discussed with the perpetrator, in the hopes of, for example, raising awareness of potential legal ramifications and the existence of unintentional abuse.

### Improving Access to Support

This category included seven intervention strategies where the EARS/SPP team provided or facilitated the victim’s engagement with support services or resources. *Legal Support* involved providing legal resources to victims and included 30 different interventions such as supporting the victim to understand legal documentation and directing them to legal aid services. *Resource Sharing* occurred when the victim was given a comprehensive guide to local resources or was directed to such services (e.g., charities); 19 such interventions were used. *Support with Tasks* included 11 interventions where the victim was provided with support for practical tasks such as organizing cleaners to visit the home or delivery of court documents. *Financial Support* occurred when the victim was provided with information and support to meet their financial needs. Seven such interventions were identified, including discussions regarding financial options and referrals to sources of emergency funding. The strategy *Support Groups* encompassed instances where victims were encouraged to attend preexisting community groups that targeted their specific need. Five such varying interventions were identified, including support groups for families with addiction, family violence, and bereavement issues. *Cultural and Religious Services* included interventions linked to the culture or religious beliefs of the victim. The eight interventions included referrals to Indigenous services and providing details of culturally appropriate emergency services. The intervention strategy *Food Resources* included four interventions to assist the victim in obtaining food. Examples include providing information about centers that offer free meals and liaising with the foodbank to reinstate a victim’s services.

### General Safety Actions

General Safety Actions included interventions designed to ensure the victim’s immediate safety. Twelve interventions, such as safety planning and encouraging the victim to pack an emergency bag were identified.

### Home Safety

This category includes intervention strategies designed to safeguard the victim’s home environment. *Housing Support* involved varying interventions (*n* = 19) to secure accommodation, including helping the victim find a new home and making a safe house referral. *Environmental Changes*, defined as any changes made to the victim’s home environment to increase safety, included five interventions, including putting bars on windows and installing an alarm system.

### Education

This category included two intervention strategies where education was provided to the victim to help end the abuse. *Elder Abuse Education* provided 12 educational interventions to victims to empower their knowledge, including explaining the cycle of violence and different types of abuse. *Mental Health and Substance Abuse Education* was used in eight interventions where the victim was educated about mental health or substance use, as it related to themselves and/or the perpetrator. Examples include educating the victim regarding their role as an enabler to the perpetrator or on the impact of substance abuse on physical health.

### Victim Care

Interventions within this category involved actions taken to provide direct care for the victim. *Physical Care* interventions (*n* = 18) aimed to improve the victim’s physical health and included organizing home care and implementing physical aids and supports in the home. *Mental Health Care* included treatment and support for the victim’s mental health needs, such as referrals to a mental health crisis team and completing intake referral forms for the victim to be seen and assessed by a psychologist. *Substance Abuse Care* included two interventions (addiction support and advising of addiction services) used in the same case to provide substance abuse–related care for the victim.

### Victim Well-Being

Three interventions within this category were identified as attempts to support the victim’s well-being. These interventions differed from mental health care in that they were not clinically or diagnostically focused but instead aimed to adjust thoughts and actions to improve the victims’ resilience and choices related to the abuse. *Emotional Support* occurred when the victim was provided with support, encouragement, or reassurance regarding their concerns. This included 23 types of interventions such as support in processing feelings of guilt and encouragement to reduce social isolation. *Reasoning with Victim* involved discussions where victims were challenged, encouraged, or otherwise reasoned with when they appeared to be making decisions that could place them in danger. These interventions (*n* = 13) included challenging the victim regarding their protectiveness of the perpetrator and encouraging the victim to renew their protection order against the perpetrator. *Development of Communication* included 17 interventions designed to help the victim develop the skills and confidence to express themselves and their needs in a healthy way, such as developing assertiveness skills and role-playing.

### Capacity Assessment

Capacity assessments were required when capacity, defined as “the ability to understand the information that is relevant to the making of a personal decision and the ability to appreciate the reasonably foreseeable consequences of the decision” (Alberta Personal Directives Act, 2000, p. 3), was in question. In such circumstances, the EARS/SPP team would arrange for the victim to have a capacity assessment completed by a trained professional. The outcomes of these assessments sometimes, for example, led to the team assisting safe family or friends, to enact either a preexisting personal directive and/or power of attorney directive.

### Involvement of Others

This category included two intervention strategies where the EARS/SPP team brought in other professionals or individuals known to the victim to assist in implementing safety measures. *Multidisciplinary Teams* interventions (*n* = 26) involved additional external services coming to work with EARS/SPP to ensure the victim’s safety. This included the EARS/SPP team joining a hospital case conference to communicate background information and current concerns, and police officers supporting EARS/SPP caseworkers on home visits. The strategy *Family and Friends* included interventions (*n* = 15) where such individuals were engaged and educated on how they could support the victim to reduce the risk of abuse. Example interventions include family meetings and organizing guardianship within the family.

### Management Strategy Efficacy

Positive outcomes were the most common results of the interventions employed by the team, occurring 384 times across 108 cases. An average of two positive outcomes occurred per case (*SD* = 2.91, range = 0–20). Unknown outcomes were the next most common, occurring 293 times across 119 cases (*M* = 1.79, *SD* = 1.66, range = 0–6). Next were interventions that could not be implemented, which occurred in 236 instances across 105 cases with an average of one blocked strategy per case (*SD* = 1.61, range = 0–8). Interventions had neutral outcomes in 177 instances across 90 cases, with an average of one per case (*SD* = 1.47, range = 0–10). Negative outcomes were the least likely, occurring 13 times across 11 cases (*M* = 0.08, *SD* = 0.31, range = 0–2).

The efficacy of the 12 intervention categories and 30 intervention strategies is displayed in Table 2. Categories Creating Barriers to Abuse and Capacity Assessment included mostly positive outcomes. At the strategy level, Supervision, Removing Access to Methods of Abuse, Physical Treatment for the perpetrator, Mental Health and Substance Abuse Education for the victim and Victim Capacity Assessment all had positive outcomes in most cases. Categories and strategies high in neutral outcomes tended to have low base rates. The category Home Safety and four intervention strategies (Social Support, Substance Abuse Support, Support with Tasks, Support Groups) were blocked from implementation in at least a third of cases, highlighting instances where improvements to how interventions are framed to victims or executed could improve case outcomes. Unknown information reached 40% or more in the General Safety Actions category and within two intervention strategies (Resource Sharing, Cultural and Religious Services), indicating difficulty in tracking outcomes. The results in Table 2 reinforce the rarity of negative outcomes (1% of cases).

## Discussion

This study builds on previous research by identifying and evaluating the risk management employed by a multidisciplinary EA prevention program. The results revealed the largest number of risk management interventions identified to date, increasing the breadth of our understanding regarding management in this area. For instance, the largest number of perpetrator interventions (designed for all types of perpetrators, not only caregivers) was identified along with the efficacy of those interventions. The three levels of intervention presented also provide greater detail than previous studies and, in conjunction with the efficacy data, can assist in sharing best practice. This level of detail also allows aspects of the program examined to be adopted without the implementation of an entirely new program. This may be of assistance in areas with limited resources, different laws or existing interventions that could be augmented by including new concepts, goals, or intervention partners like trusted family or friends.

In this study, management adhered to best practice by involving multiple professional types (Pillemer et al., 2016) and focusing on victim and perpetrator interventions (Storey, 2020). Perpetrator intervention has been generally overlooked in previous research, with Alon and Berg-Warman (2014) only identifying one perpetrator strategy and Nahmiash and Reis (2001) only examining caregivers. Management also included a broad spectrum of interventions, recognizing that intervention must be holistic, addressing perpetrator risk/need and victim vulnerability/need. Using interventions targeted at risk and vulnerability to mitigate EA risk is also best practice and an objective/product of risk assessment tools (Hart, 2001; *The Harm to Older Persons Evaluation* or *HOPE*, Storey et al., 2021, formerly the *EARLI*). The nature of the interventions (e.g., victim well-being, education) also suggests that EARS/SPP was using the empowerment model, which is supported by the research literature (Nahmiash & Reis, 2001).

The strategy most frequency recommended/implemented was Legal Support, followed by Multidisciplinary Teams. Although it is sometimes the case that victims do not wish to proceed with criminal justice remedies, Legal Support herein had a much broader scope. In fact, Legal Support contained the largest number of interventions, including explaining legal rights and issues around power of attorney. As such, it is important for those managing EA cases to have adequate legal training or access to legal services well versed in matters related to older adults and EA. Although positive outcomes were most common for Legal Support, this strategy and others within the category Improving Access to Support, had high levels of unknown outcomes and outcomes that could not be implemented (abbreviated to *Could Not Implement* or CNI). Two other support strategies, Housing Support and Social Support, also had high unknown and CNI outcomes. This suggests that there was a level of difficulty in assessing the efficacy of support-related management, perhaps because these offers of assistance have less defined or immediate outcomes. Results may also reflect a reluctance among victims and perpetrators to accept support. This may be related to attitudes around help-seeking (e.g., shame), the victim’s attitudes toward the perpetrator (e.g., feeling responsible for them), a victim’s perception of what is abusive or the perception that accepting support will make things worse (e.g., loss of relationship, increase abuse). Increased education may help to reduce such barriers.

Multidisciplinary teams were the second most frequently used strategy and had the second highest number of interventions. The category Involvement of Others, which includes this strategy, most often had positive outcomes, followed by unknown outcomes. Continued support for such teams and cross-professional information sharing are important. To improve the evaluation of this strategy, future research should examine records from all professionals involved in a case or survey those professionals.

The overall results related to efficacy were highly promising. Positive outcomes were the most frequent and negative outcomes the least. These results underline how important and helpful it is for professionals to engage in risk management. It is critical that professionals continue to have the time and resources to learn about and engage in risk management because when they do, the results are overwhelmingly positive.

Strategies that showed the most positive outcomes were within the category Creating Barriers to Abuse. Supervision interventions that related to restrictions on the perpetrator such as legal conditions or bans were frequently positive. Removing Access to Abusive Methods such as bank accounts and positions of authority also resulted in mostly positive outcomes. However, as a proportion of the total number of interventions used, these were not common. This could therefore be a place where a change in practice could have a substantial impact on case outcomes. To increase use, it will be important to emphasize that harsher punishment or a reliance on the legal system may not be the reason that this category is effective. For instance, in one case, multiple legal interventions, including an emergency protection order and a peace bond (restraining order), had positive outcomes, including an end to the abusive behavior and a victim who felt safe and happy. In this case, the legal conditions were specific to the perpetrator’s risk factors (i.e., mental health problems, substance abuse, and dependence on the victim) and thus targeted empirically supported needs related to EA perpetration. Therefore, it was not the severity of the punishment but its specificity in targeting offender needs that likely had the desired positive effect. Furthermore, following the legal intervention, the EARS/SPP caseworker was able to conduct unannounced follow-ups and, as a result, identified the perpetrator’s relapse early and was able to again implement risk/need-focused interventions to avoid deterioration of the progress made.

Another intervention that had positive outcomes in most cases was capacity assessment. Although not utilized frequently due to the gravity of the potential outcome, its implementation demonstrated that it could be strategic and versatile as an intervention to assist victims. Capacity assessments were sometimes used to guide action by providing clarity regarding the presence of certain behaviors. In one case, a victim who was making relatively dangerous choices regarding association, and was victimized by an associate was given a capacity assessment that uncovered memory problems, allowing the root cause of the dangerous behavior to be identified and addressed. Capacity assessments were also used to return victim agency and create distance between the victim and perpetrator. In another case, a capacity assessment proved that the victim was competent and resulted in the perpetrator being removed as their agent. With increasing instances of dementia (Alzheimer’s Society, 2017) and cognitive impairment being a significant risk factor for EA (Storey, 2020), it is critical that we understand and effectively use this strategy.

The results also identify ways to improve the risk management process. CNI and Unknown outcomes followed positive outcomes as the next most frequent. CNI outcomes were highest for Home Safety and Perpetrator Treatment. Capturing all of the reasons for this and solutions to decreasing CNI in these areas will require further investigation. Some reasons identified for Home Safety CNI included a lack of resources (e.g., safe house was full) and victim refusal; Perpetrator Treatment was also often CNI due to refusal. The lack of resources suggests a need for better government and community resourcing to support victims of EA. Promoting service acceptance by victims and perpetrators could be ameliorated by using psychological interventions such as motivational interviewing, which has been shown to be effective in promoting change among EA victims (Mariam et al., 2015). It is possible that victims and perpetrators may not understand the consequences of engaging with services, thinking only that they will lose autonomy once they enter the system. Education around their rights could reduce service refusal. Refusal of services by perpetrators could be remedied through increased use of legal interventions that make treatment a legal condition, where charges have been brought. Nevertheless, the need for evidence-based practice is imperative. Interventions can be traumatic for individuals, raising difficult emotions and memories (Douglass, 2005). Prior to challenging service refusal, we must ensure that the cost of intervention is balanced with the efficacy of the intervention.

Unknown outcomes were highest for General Safety, Improving Access to Support, and Involvement of Others. Two reasons seem plausible for this. First, some of the strategies included in these categories (e.g., support groups, resource sharing) require time to produce and identify measurable impact. Thus, impact was not identifiable prior to case closure. Second, some interventions do not lend themselves well to discrete outcomes or outcomes that would be recorded in case notes. For example, General Safety included interventions like developing a safety plan and packing an emergency bag. The impact of both interventions would only be realized if a major incident occurred. Alternate research methodologies, such as exit interviews with victims querying intervention efficacy, or alterations to recording procedures, where recording efficacy is encouraged, may reduce unknown outcomes.

Some limitations and strengths should be considered when interpreting this study’s results. The examination of a single Canadian service may limit generalizability. To broaden the utility of the work, the results were primarily presented at the category and strategy levels, as broader concepts related to risk management are more likely to be generalizable. Ethical considerations meant that there was no control group; this has been a criticism of related research (Fearing et al., 2017) and is one of the reasons that case record analysis is the most common design (Rizzo et al., 2015). Long-term follow-up data were not available. However, because each case and the outcome of each intervention were examined from case reporting to conclusion, instances of reoffense within that time were captured.

Several aspects of this study’s design reduced the possibility of bias and addressed the pitfalls raised by Rizzo and colleagues (2015). First, consecutive cases over a 27-month period were used, providing a representative sample of the cases encountered by EARS/SPP and avoiding sampling bias. Second, because data were retrospective, EARS/SPP workers were unaware of the research while recording their notes. Third, by examining actual practice (rather than using surveys or interviews), recall bias was eliminated. Rater reliability was excellent, suggesting limited coding bias. The study also had a relatively large sample size.

This study identified many novel interventions for EA and provided efficacy data for the widest range of EA interventions to date. Results showed a high prevalence of positive outcomes and identified several areas where policy, practice, and methodological changes could reduce unknown and CNI outcomes, thereby increasing positive outcomes. Findings can be used to advance intervention programs and support practitioners in the management of EA.
